# Scope, training, and systemic challenges of family medicine in South Asia: Perspectives of general practitioners and family physicians

**DOI:** 10.12669/pjms.42.(ICON26).15697

**Published:** 2026-04

**Authors:** Faridah Amir Ali, Raman Kumar, Kabir Ahmed, Sankha Randenikumara, Mannawar Sarwar Ali

**Affiliations:** 1 Dr. Faridah Amir Ali, Director Indus College of Family Medicine and Public Health (IUHS), Indus Hospital and Health Network, Pakistan; 2 Dr. Raman Kumar, Director Institute of Family Medicine and Primary Care (iFMPC), Uttar Pradesh, India; 3 Dr. Kabir Ahmed, Head of Family Medicine Unico Hospital, Dhaka, Bangladesh; 4 Dr. Sankha Randenikumara, Chief Family Physician, The Family Health Clinic, Galle, Sri Lanka; 5 Mr. Mannawar Sarwar Ali, Senior Officer Office of Research, Innovation & Commercialization, Indus Hospital and Health Network, Pakistan

**Keywords:** Career Pathways, Family Medicine, General Practitioners, Primary Health Care, South Asia, Postgraduate Medical Education, Workforce Development

## Abstract

**Background and Objective:**

Family Medicine (FM) specialty central to primary-care and universal health coverage, remains underdeveloped with limited structured training, regulatory support, and career prospects in South Asia. This study assessed perceptions of general practitioners (GPs) and family physicians (FPs) regarding its scope, training opportunities, and systemic challenges.

**Methodology:**

A cross-sectional online survey was conducted between March – December, 2024 amongst physicians of Pakistan, India, Bangladesh, and Sri Lanka. Eligible participants included GPs, postgraduate FM trainees, and FPs from Pakistan, India, Bangladesh, and Sri Lanka. A structured questionnaire gleaned information on demographics, training, continuous medical education (CME) access, clinical guidelines, research, and career opportunities. Data were analyzed using SPSS-version-26.

**Results:**

Of 246 respondents (53% Pakistan, 26% India, 15% Bangladesh, 7% Sri Lanka), 58% were females and 42% males. Overall, 63% reported access to FM training with greater satisfaction illustrated by Pakistan. Access to CME and clinical guidelines was 58% and 23%, respectively. More Pakistani participants perceived improved scope (*p*<0.001), public awareness (*p=*0.033), and career opportunities (*p=*0.038). Majority 87%, suggested reserving the term “Family Physician” for those with postgraduate qualifications.

**Conclusion:**

FM in South Asia despite some progress remains constrained by capacity building and weak career recognition. Strengthening academic departments, postgraduate programs, and career pathways is vital to advance PHC in the region.

## INTRODUCTION

Primary healthcare (PHC) is the foundation of effective health care systems and an essential step towards universal health coverage (UHC).^1^ Globally, Family Medicine (FM) has been the core specialty of primary care for delivering continuous, person-centered care across the lifespan. Evidence links robust FM systems to improved health outcomes, equity, and cost-efficiency.^2^-^5^

In South Asia, however, primary care and FM remain underdeveloped.^6^ Countries such as Pakistan, India, Bangladesh, and Sri Lanka often rely on general practitioners (GPs) who are medical graduates without postgraduate qualification or training to deliver front line primary care services.^7^ In many cases, these practitioners represent more than 70% of the medical workforce involved in primary care.^8^ FM as a specialty is relatively new in the region with limited postgraduate programs and inclusion in some medical curricula and structured training programs.^6^,^9^ Weak regulatory support, limited career pathways, insufficient continuous medical education (CME) opportunities and trainings, and shortage of qualified consultants and trainers further enhance these gaps.^8^ Shortage of trained family physicians at primary care facilities raises concerns regarding comprehensiveness of care. Moreover, there is a lack of a specific regulatory body for general practice, as well as a deficit of established local clinical guidelines. Thus, general practitioners are medical graduates without postgraduate qualifications or formal training and often work in situations akin to professional isolation,^7^ despite in many cases, these practitioners representing more than 70% of the medical workforce involved in primary care.^8^ Despite these challenges, there is a growing interest in strengthening primary care in the region. Governments and academic institutions are beginning to recognize the strategic importance of the FM specialty in primary care.^1^ This evolving landscape presents an opportunity to assess how front-line providers perceive the specialty and identify enablers for its growth.

International evidence indicates that investment in trainings, guidelines development, and policy recognition can strengthen FM and improve PHC performance.^8^,^10^ Therefore, an understanding of perspectives of practitioners on current state and future growth of the specialty is crucial to guiding policy reforms. To date, no large-scale cross-country study has explored the perceptions of GPs and family physicians and trainees in South Asia on the scope, training availability, and systemic challenges of the specialty. Understanding these insights is essential for policy development, institutional planning, and implementation of reforms that are aligned with provider realities. This study therefore aimed to assess perceptions of GPs and family physicians in South Asia regarding the scope, progress, training and CME accessibility, and strategies for strengthening FM as a recognized specialty in the region.

## METHODOLOGY

A cross-sectional online survey was conducted amongst physicians of Pakistan, India, Bangladesh, and Sri Lanka between March and December 2024. The participants were attendees of the World Organization of Family Doctors (WONCA) South Asia Conference 2024 where the prospective participants were oriented and invited to participate in the study. Survey was then conducted via emails, WhatsApp groups, and social media. The sample size 296 was calculated by using Open epi software with 95% confidence level, 5% precision, and an expected professional satisfaction proportion of 74% among family physicians.

### Ethical Approval:

Ethical approval was granted by the Indus Hospital & Health Network Institutional Review Board (IHHN_IRB_2024_04_004; dated April 17, 2024).

Eligible participants selected via non-probability consecutive sampling technique were licensed GPs, Family Physicians, postgraduate FM Trainees and Consultants. Specialists from other disciplines and those not proficient in English were excluded. Electronic informed consent was obtained from all participants.

The questionnaire was developed via a pilot survey of 30 participants (12% of sample size) using open-ended questions. Responses were analyzed using inductive content analysis to identify recurring codes and categories, and converted into a structured questionnaire with response options items also derived from pilot data. Key thematic areas of the questionnaire were training pathways, undergraduate teaching, job opportunities, leadership, government recognition, and strengthening primary health care through FM. The questionnaire was then shared with investigators from across South Asia to adapt it for local relevance and content validity. This finalized structured questionnaire gleaned participant information on demographics, professional background, training exposure, clinical guidelines, continuous medical education (CME), perceptions of career opportunities, research engagement, financial satisfaction, public awareness, availability of FM consultants and strategies for strengthen FM specialty.

Data were analyzed using SPSS version 26. Descriptive statistics were used to summarize categorical variables as frequencies and percentages. Continuous variables were reported as medians with interquartile ranges. As highest participation was from Pakistan, a comparative analysis between participants from Pakistan and those from other South Asian countries was performed using chi-square tests for categorical variables and Mann-Whitney U tests for continuous variables.

## RESULTS

### Participant characteristics:

A total of 246 participants completed the survey. Of these, 58% were females and 42% were males with majority at ≤ 40 years of age (56%), followed by 41-59 years (35%) and ≥ 60 years 9%. Majority were from Pakistan 131(53%), followed by India 63(26%), Bangladesh 36(15%), and Sri Lanka 16(7%). Professional designations included GPs, family physician 29%, postgraduate trainee 24%, medical officer 19%, consultant family physician 18%, and others 10%. Median years of practice were 10. Approximately 88% were currently practicing, 12% were non-practicing and into profession such as academia, social sector and institutional administration.

One-third (32%) lacked post graduate qualification while more than half either had a one-year diploma or training in general practice; or were post graduate doctors. The other most common post graduate qualifications were: Pakistani’s membership of College of Physicians and Surgeons Pakistan (MCPS) 33%, Diplomat National Board (DNB) in India 52%, and Membership of College of General Practitioners (MCGP) in Sri Lanka 38%. Half of the GPs in Bangladesh did not have a post graduate qualification while 22% had attended a course or one year diploma/training. Approximately six percent of all participants had a membership of Royal College of General Practitioners (MRCGP) international qualification across South Asia.

### Training and Trainer availability:

Access to structured FM training was reported by 63% of participants, though only 37% had training available within their workplace. Training programs were more available in other South Asian countries compared to Pakistan (70% vs. 56%, p=0.034, Table-I). However, GPs in Pakistan were more likely to be satisfied with the available training programs and reported a higher presence of qualified consultants on site than participants from other South Asian countries (46% vs. 29%, p=0.006). Half (50%) of all GPs reported having no trained FM consultants at their workplace (Table-I).

**Table-I:** Comparison of training pathways and scope of family medicine between Pakistan and other South Asian countries

Questions	Responses	Pakistan	South Asian other than Pakistan	Total	P-value
		n=131	n=115	n=246	
Training in Family Medicine					
Structured training pathway in Family Medicine	Yes	74(56)	80(70)	154(63)	0.034^ⱡ^
	No	57(44)	35(30)	92(37)	
Structured Family Medicine training program at institution/ practice	Yes	52(40)	40(35)	92(37)	0.427*^ⱡ^
	No	79(60)	75(65)	154(63)	
Satisfaction with Family Medicine training programs in their country	Yes	60(46)	33(29)	93(38)	0.006*^ⱡ^
	No	71(54)	82(71)	153(62)	
Ease of access to Family Medicine training pathways in their country	Strongly Agree	9(7)	9(8)	18(7)	0.432^Ŧ^
	Agree	54(41)	36(31)	90(37)	
	Not Sure	26(20)	33(29)	59(24)	
	Disagree	38(29)	33(29)	71(29)	
	Strongly Disagree	4(3)	4(3)	8(3)	
Availability of trained Family Medicine consultants at workplace	1-2	33(25)	35(30)	68(28)	<0.001**
	>2	43(33)	12(10)	55(22)	
	None	55(42)	68(59)	123(50)	
Availability of Continuous Medical Education at Workplace/Locality	Yes	72(55)	70(61)	142(58)	0.394^ⱡ^
	No	59(45)	45(39)	104(42)	
Desire to Take training/courses in research writing	Yes	88(67)	84(73)	172(70)	0.317^ⱡ^
	No	43(33)	31(27)	74(30)
Career Opportunities and Scope of Family Medicine					
Career opportunities for Postgraduate Family Physicians vs. General Practitioners	Yes	72(55)	48(42)	120(49)	0.038^ⱡ^
	No	59(45)	67(58)	126(51)	
Satisfaction with the Financial Compensation of Family Physicians	Yes	12(9)	7(6)	19(8)	0.368^ⱡ^
	No	119(91)	108(94)	227(92)	
Public awareness of Family Medicine over the last 5 Years	Yes	62(47)	39(34)	101(41)	0.033^ⱡ^
	No	69(53)	76(66)	145(59)	
Scope of Family Medicine over the last 5 years	Yes	111(85)	76(66)	187(76)	0.001*^ⱡ^
	No	20(15)	39(34)	59(24)	

* P-value<0.05,

** P-value<0.0001, ⱡ Pearson Chi Square test, Ŧ Fisher’s Exact Test.

### Career opportunities and scope of Family Medicine:

More than half respondents, 51%, believed that there were not enough career opportunities for trained or qualified family physicians or were unsure about available options (p=0.038). However, 76% felt that the scope of FM had increased in their region over the past five years (Table-I).

Interestingly, GPs from Pakistan were more likely than those from other South Asian countries to perceive an increase in career opportunities (p=0.038), scope (p < 0.001), and public awareness (p=0.033) of FM in the last five years. Despite this, more than half of all participants felt that public awareness has not increased, and majority recommended media campaigns and induction of more qualified family physicians in both public and private sectors (Table-I). A good majority 92% were also dissatisfied or uncertain about the financial compensation for GPs.

### CME, clinical guidelines and Research Engagement:

Although 42% reported no continuing medical education (CME) activities, 85% further said they would prefer virtual CME if possible. Only 23% reported consistent availability of clinical guidelines in their region, with no significant difference between Pakistan and other countries with a 47% favoring guideline development jointly government regulatory bodies and family physicians. Where clinical growth opportunities are scarce, other avenues such as research opportunities were also reported by 64% as not available to them, predominantly publications, although seven percent considered research paper writing as easy whereas 70% expressed interest in receiving training in research. Publications were higher amongst Pakistani participants 29% compared to combined publications from other South Asian countries 43% (p=0.018) (Table-II).

**Table-II:** Continuous medical education (CME), clinical guidelines, and research engagement for General practitioners/Family Physicians of South Asia.

Question	Responses	Pakistan	South Asian other than Pakistan	Total	P value
		n=131	n=115	n=246	
Availability of clinical guidelines for Primary Care practice	Yes	30(23)	27(23)	57(23)	0.612^ⱡ^
	To Some Extent	60(46)	46(40)	106(43)	
	No	41(31)	42(37)	83(34)	
Who should develop local Primary Care Guidelines?	Regulatory bodies (government) through family physicians	63(48)	53(46)	116(47)	0.830^ⱡ^
	Regulatory bodies through various specialists	13(10)	11(10)	24(10)	
	Family medicine consultants	51(39)	38(33)	89(36)	
	Other	4(3)	13(11)	17(7)	
Research Publications in National/International Journals	Yes	38(29)	50(43)	88(36)	0.018^ⱡ^
	No	93(71)	65(57)	158(64)	
Is writing a primary care research paper easy?	Strongly Agree	9(7)	8(7)	17(7)	0.412^Ŧ^
	Agree	29(22)	32(28)	61(25)	
	Not Sure	60(46)	48(42)	108(44)	
	Disagree	27(21)	26(23)	53(22)	
	Strongly Disagree	6(5)	1(1)	7(3)	
Desire to take research training/ course in research writing	Yes	88(67)	84(73)	172(70)	0.317^ⱡ^
	No	43(33)	31(27)	74(30)	

*P-value<0.05, **P-value<0.0001, ⱡ Pearson Chi Square test, Ŧ Fisher’s Exact Test.

### Perceptions on improving quality of general practice:

Respondents from Pakistan and other South Asian countries emphasized the need to expand training pathways 66% vs. 59%. and make FM a mandatory part of the undergraduate curriculum 56% vs. 63%. Participants from other South Asian countries showed stronger agreement 61% that establishing mandatory FM departments in academic institutions is essential, compared to 44% of Pakistani respondents.

Participants emphasized the need for more job opportunities locally; albeit the need for international prospect was not undermined. Suggestions included stronger leadership, better government receptiveness, and greater recognition of FM as a cost-effective primary health care model. A few respondents suggested restricting general practice to formally trained family physicians.

### Suggestions to strengthen primary care through Family Physicians:

**Fig.1:**
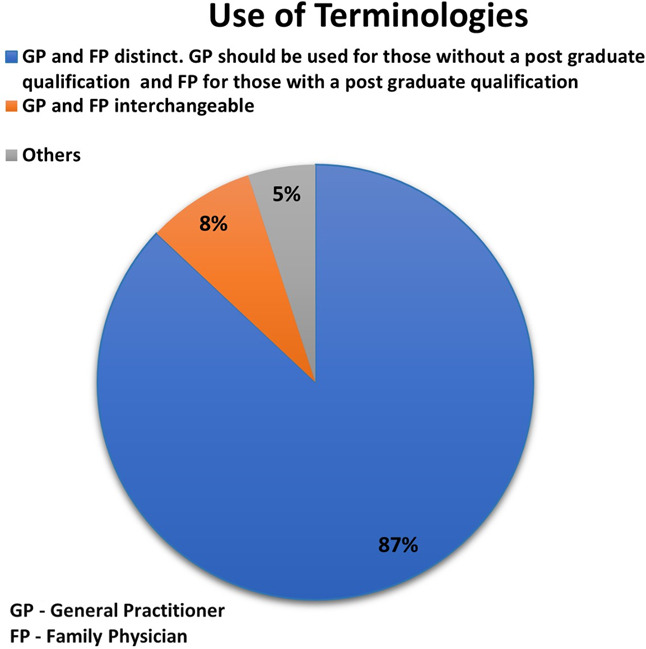
Suggested use of terminologies by participants.

Most respondents agreed that FM should serve as the first point of contact in the public sector and that primary health care services should be formally recognized and accredited by institutions and regulatory bodies. A majority 87% believed that the term *General Practitioner* should apply to those without a postgraduate qualification, while *Family Physician* should be reserved for those with postgraduate training in FM (Fig-1). Many recommended creating a dedicated cadre of family physicians in the government sector, providing incentives through health insurance, and ensuring supervision of primary care units by qualified family physicians. Several also emphasized the role of tele-health services in extending Family Medicine to rural populations.

## DISCUSSION

Studies on various dimension of FM abound but this study is one of the first multi-country surveys to examine the perceptions of front-line physicians on FM development in primary care in South Asia. Opportunities and accessibility for post graduate training and availability of trainers continue to limit the growth of FM across SAR.^11^ The reliance on GPs, without postgraduate qualifications is consistent with earlier studies from Pakistan and India, which reported that over 70% of primary care is delivered by non-specialist practitioners.^7^,^9^ Satisfaction with training, and availability of FM Consultants in Pakistan may reflect the mandatory presence of FM departments in medical colleges and inclusion of specialty undergraduate curricula, enabling greater opportunities for training and career advancement.^12^ However, the persistent lack of adequate supervision and limited structured postgraduate training across all participating countries is consistent with findings from earlier studies conducted in Pakistan and India.^8^,^9^ Although initiatives such as Membership College of Physicians and Surgeons of Pakistan (MCPS), Diplomat National Board (DNB) in India, Membership College of General Practitioners (MCGP) in Sri Lanka and, Membership Royal College of General Practitioners (MRCGP) international in SAR represent progress, their impact remains constrained by inconsistent governmental recognition, adversely contributing to ongoing professional isolation. Similar challenges have been documented in 25 countries of Asia-Pacific region, where only about half of surveyed institutions offer clinical postgraduate programs, and even fewer provide postgraduate research opportunities.^13^ A lack of qualified trainers, as reported in Southeast Asia, further reflects these same challenges.^14^ Collectively, these patterns demonstrate longstanding systemic neglect of primary care training, in contrast with high-income countries where postgraduate reforms since the 1960s have established general practice as a recognized specialty.^15^ Strengthening academic departments and investing in trainer development are therefore essential to scale FM in South Asia.

Primary care continues to be undervalued in comparison to hospital-based specialties, leaving limited career prospects and professional recognition for trained family physicians. This aligns with previous studies from Pakistan and India documenting dissatisfaction with compensation and restricted career pathways.^15^,^16^ Nonetheless, respondents’ perception of expanding scope and awareness may indicate early policy-level progress, consistent with recent undergraduate reforms and emerging training opportunities.^6^,^17^,^18^ Evidence from SAR suggests that while the profile of FM is improving, structural barriers particularly lack of specialist recognition in public health systems continue to restrict growth.^15^

Public awareness also remains limited, echoing prior recommendations for media engagement and advocacy to improve recognition of the specialty.^19^ Furthermore, debates around nomenclature (general practitioner vs. family physician) reflect ongoing efforts to define professional identity. Internationally, similar distinctions have evolved in parallel with postgraduate reforms.^15^

Gaps in continuing professional education and lack of local clinical guidelines further constrain the discipline. Earlier reviews noted the limited scope of CME in the region, with online delivery recommended as a practical solution for resource-limited settings.^1^ The lack of context-specific primary care guidelines adds to fragmented service delivery, reflecting wider governance and integration challenges.^19^ Weak engagement in research has also been documented, attributed to scarce mentorship and institutional infrastructure.^8^ Moreover, the ground reality is that a vast majority of practicing primary care physicians in SAR do not undergo formal structured postgraduate training, hence, their capacity to conduct research is also limited. Research remains a neglected area even in the four-year structured postgraduate residency programs across Asia.^13^,^14^ Comparable shortcomings in CME, guideline development, and research networks in Southeast Asia,^1^ highlight that these challenges extend beyond SAR to other middle-income health systems. Thus utilization and application of research-informed clinical guidelines generated from the actual context of practice and research, are directly linked with standards of care. Due to the deficiency of this component, the quality of care is compromised.^1^ Interestingly, strong practitioner interest in research training highlights an opportunity for capacity-building, if formal pathways are supported.

Respondents from across SAR emphasized that strengthening FM requires both educational reforms and structural support within health systems. Up-scaling post graduate training pathways, embedding FM as a mandatory component of undergraduate curricula, and establishing academic departments were identified as key enablers. The call for improved career opportunities, both locally and abroad, echoes broader regional concerns regarding limited recognition of family physicians within public health care systems.^15^ Policy-level recommendations included creating a dedicated cadre of family physicians in government services, linking incentives to health insurance schemes, and formally accrediting primary care facilities. Supervision of front-line units by trained family physicians and the integration of tele-health to extend services to underserved areas were also highlighted, aligning with the vision of World Health Organization (WHO) of family practice as a cost-effective model for achieving universal health coverage.^1^

### Limitations:

This study provides regionally diverse insights of GPs across South Asia, but its limited sample size of participants largely conference-linked and digitally connected physicians, potentially overrepresented those more engaged in FM development cannot be generalized to the entire region. Unequal country representation, particularly higher proportion from Pakistan could have influenced comparative findings. Nevertheless, the study is strengthened by its multi-country scope, inclusion of multiple practice domains, and its contribution to a largely under-researched area of FM in South Asia.

## CONCLUSION

Family medicine in South Asia was perceived to be is at a critical juncture with a weak infrastructure for both clinical practice and training. Strengthening Family Medicine is essential to achieve UHC and address the growing burden of chronic diseases in South Asia. Building sustainable PHC systems requires combined efforts of governments and institutions for inclusion of FM in the overall health systems as an integral component; not just as a specialty providing health care but also on the development and enhancement of the specialty itself.

### Authors` Contribution:

**FA** conceived, designed, literature review, data collection, discussion, conclusion, reviewed, manuscript writing, and gave final approval of the manuscript.

**RK, KA, SR** literature review, manuscript writing.

**MS** statistical analysis, result writing, methodology, table & graph and finalization of script and also responsible for the accuracy of the study.
